# Effects of dependence in high-dimensional multiple testing problems

**DOI:** 10.1186/1471-2105-9-114

**Published:** 2008-02-25

**Authors:** Kyung In Kim, Mark A van de Wiel

**Affiliations:** 1 Department of Mathematics and Computer Science, Eindhoven University of Technology, Eindhoven, The Netherlands; 2 Department of Mathematics, Vrije Universiteit Amsterdam, Amsterdam, The Netherlands; 3 Department of Pathology & Department of Biostatistics, VU University Medical Center, Amsterdam, The Netherlands

## Abstract

**Background:**

We consider effects of dependence among variables of high-dimensional data in multiple hypothesis testing problems, in particular the False Discovery Rate (FDR) control procedures. Recent simulation studies consider only simple correlation structures among variables, which is hardly inspired by real data features. Our aim is to systematically study effects of several network features like sparsity and correlation strength by imposing dependence structures among variables using random correlation matrices.

**Results:**

We study the robustness against dependence of several FDR procedures that are popular in microarray studies, such as Benjamin-Hochberg FDR, Storey's q-value, SAM and resampling based FDR procedures. False Non-discovery Rates and estimates of the number of null hypotheses are computed from those methods and compared. Our simulation study shows that methods such as SAM and the q-value do not adequately control the FDR to the level claimed under dependence conditions. On the other hand, the adaptive Benjamini-Hochberg procedure seems to be most robust while remaining conservative. Finally, the estimates of the number of true null hypotheses under various dependence conditions are variable.

**Conclusion:**

We discuss a new method for efficient guided simulation of dependent data, which satisfy imposed network constraints as conditional independence structures. Our simulation set-up allows for a structural study of the effect of dependencies on multiple testing criterions and is useful for testing a potentially new method on *π*_0 _or FDR estimation in a dependency context.

## Background

Scientists regularly face multiple testing of a large number of hypotheses nowadays. Typically in microarray data, one performs hypothesis testing for each gene and the number of genes is usually more than thousands. In this situation, direct application of single hypothesis testing thousands times produces a large number of false discoveries. Hence, alternative testing criterions for controlling errors of false discoveries have been introduced.

It is widely recognized that dependencies are omnipresent in many high-throughput studies. Such dependencies may be regulatory or functional as in gene pathways, but also spatial such as in SNP or DNA copy number arrays because of the genomic order. Although attempts to infer such interactions from data have been made, it is a notoriously difficult problem. Usually solutions focus on some modules with relatively few elements and many samples, in particular for model organisms (see e.g. [1]). With this in mind, one prefers multiple testing methods that are robust to several degrees of dependency in these network-type data. Therefore, we set out to develop a simulation program that allows us testing any multiple testing method for its robustness with respect to dependency parameters using realistic nested network structures.

One of the most widely used multiple testing criterions for controlling errors of false discoveries is False Discovery Rate (FDR). FDR is introduced in Benjamini et al. [2] and is defined as the expected proportion of the number of falsely rejected hypotheses among total number of rejected hypotheses. Since in most cases, underlying distributions of data are unknown, there are several implementations of FDR under different assumptions.

Benjamini et al. [2] first suggest an implementation of FDR by a linear step up approach. For an *m *hypotheses multiple testing problem with *m*_0 _true null hypotheses, the Benjamini-Hochberg (BH) procedure finds maximal *k *such that *p*_(*k*) _≤ *γ*(*k/m*) where *k *= 1,..., *m*, *p*_(*k*)_'s are observed ordered *p*-values and *γ *is prespecified level of significance. The BH procedure is known to control

(1) $$\text{FDR}\leq \frac{m_{0}}{m}\gamma =\pi_{0}\gamma \leq \gamma .$$

under independence assumption of test statistics. Later, Bejamini and Yekutieli [3] prove the BH procedure controls under positive regression dependency condition and they introduce a modification of the above procedure to control arbitrary dependence circumstances (BY). Storey [4] introduces the positive false discovery rate (pFDR) and the *q*-value. pFDR is known to control error rate under the clumpy dependency condition [5]. Significance Analysis of Microarray (SAM) is developed on the purpose of statistical analysis of microarray data [6]. SAM FDR is known to estimate the expected number of false discoveries over the observed number of total rejections under the complete null hypothesis [7].

In (1), there still remains some space of improvement for tighter control if we know *π*_0_. Adaptive procedures are developed to gain more power by estimating *π*_0 _in this sense. To estimate *π*_0_, Storey et al. [5] use the fact that independent null *p*-values are distributed uniformly on [0, 1] and then plug the estimator ${\hat{\pi }}_{0}$ into the FDR-estimator. Benjamini et al. [8] estimate *m*_0 _in a two-stage adaptive control of FDR (ABH). Under the assumption of independence of test statistics, they show the ABH procedure controls nominal significance level. Careful simulation studies on the comparison of performance of *π*_0 _estimation methods are done by Black [9] and Langaas et al. [10]. Black [9] notes that the violation of uniformity of *p*-values due to the presence of non-null cases could bias estimates of *π*_0 _upward.

Recently, several works incorporates correlations among test statistics to estimate FDR. Resampling based approaches are immediate in utilizing sample correlation structure [11]. However, since it is difficult to resample from the true null and the false null distributions separately, it is common to assume the complete null hypothesis and set the number of true discoveries fixed in order to estimate FDR conservatively, as is shown in the resampling based method of Yekutieli and his coworkers [12,13]. Since the procedures mentioned above are often used, we would like to study validity of those procedures under fairly general dependence circumstances and how correlations among test statistics affect results of FDR multiple testings. Also, we would like to examine effects of violation of independence of *p*-values on *π*_0 _estimations. Hence, designing general dependence conditions is our main concern. In previous works, for convenience of simulations, data are often assumed multivariate normally distributed. Typically in microarray data analysis, equi-correlated normal structures such as single pairwise correlation matrices or block diagonal matrices with a single pairwise correlation in each block are considered [14,15].

Equi-correlated structures are easy to understand and implement. Moreover, control of dependence conditions is easy by increasing or decreasing single correlations. But they are hardly regarded to represent reality. Random correlation matrices are more realistic candidates, because they reflect heterogeneity between the correlations. However, since the class of random correlation matrices is too large, multiple testing results generated from two arbitrary random correlation matrices are difficult to compare.

Therefore, we propose constrained random correlation matrices to reflect the generality of random correlations and the comparability like equi-correlation models to simulation studies. For simulation studies, we generate a sequence of random correlation matrices and as constraints we impose conditional independence structures on the random correlation matrices in a 'nested' way. Then the sequence of random correlation matrices is ordered in terms of a dependence parameter and we control the strength of dependence by the dependence parameter. An alternative, non-nested, approach is discussed by Jung et al. [16] who simulate multivariate normal test statistics while conserving the correlation structure as present in the data in an asymptotic sense.

In our simulation results, we show how the dependence parameter can serve as a measure of FDR behavior under correlation-based dependence conditions. We prove that this dependence parameter is in fact strongly related to the variance of pairwise correlations. Using this structural simulation setting, we compare the performance of several FDR estimating methods.

## Results

We illustrate simulation results. Here, we consider two cases for the proportion of true null hypotheses: *π*_0 _= 0.8 and *π*_0 _= 0.95. Both cases show similar results, so we focus on the first case. For *π*_0 _= 0.95, we refer to Figure S12-S14 [see Additional file 1]. We do not take into account for small *π*_0_'s because in high-dimensional data with thousands hypotheses one is usually interested in the case when only small portions of total hypotheses are truly significant. We generate 16 correlation matrices Σ based on 16 edge densities, which are the proportions of non-zero partial correlations over all possible pairs of partial correlations, 0, 0.05, 0.1, 0.15, 0.2, 0.25, 0.3, 0.35, 0.4, 0.45, 0.5, 0.6, 0.7, 0.8, 0.9, 1. Note for a nested sequence of random correlation matrices, we use one initial *Z *matrix (see Algorithm 2) for each *π*_0_. The total number of hypotheses is set to *m *= 1000 and sample sizes for *X *and *Y *are 10 each. The number of resamples to compute average FDR in (5) are *B *= 2000. The fixed true difference is chosen to have 80% power for individual two group *t*-statistic when FDR significance level is 0.1 under independence assumption.

**Figure 1:**
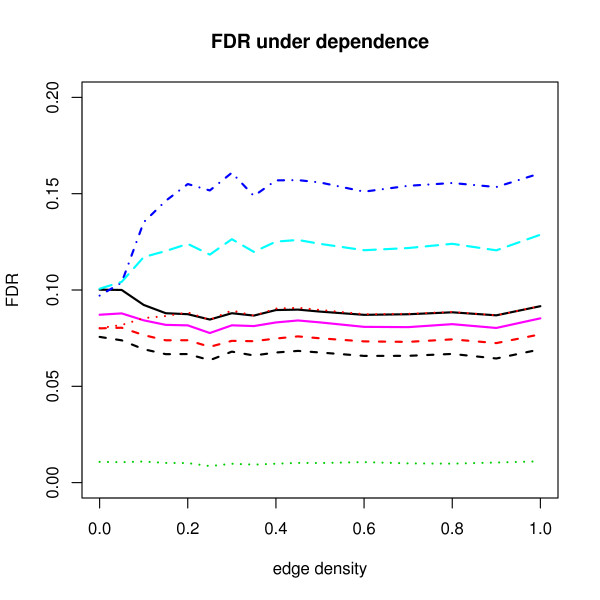
**Average FDR results under dependence when *π*_0 _= 0.8**. The *x*-axis corresponds to the proportion of edges in the network and the *y*-axis corresponds to FDR estimates for various procedures. Testing cut-off *c *is tuned such that true FDR is 0.1 under independence. FDR(*c*) (solid black) represents true FDR values in terms of (5) using the fixed *c*. The FDR procedures and corresponding lines in this figure are the following ones: BH (dashed red), BY (dotted green), SAM (dot-dashed blue), Qvalue (dashed cyan), ABH (purple), the upper limit RBH (dashed black), the point RBH (dotted red).

Figure 1 shows the FDR results under dependence when *π*_0 _= 0.8. Nominal significance level *γ *is 0.1. The black solid line represents reference FDR results using (5). Under independence, FDR(*c*_0.1_) = 0.1 as expected by the law of large number. But it decreases to around 0.085 when the edge density increases to 0.25 and then it is flatten around at 0.087. The results of SAM and Qvalue seem to overestimate FDR and these increase to 0.16 and 0.13, respectively. On the other hand, BH, ABH, RBH procedures seem to be conservative under dependence. As in (1), under independence, BH procedure controls FDR at level 0.08 = *π*_0_*γ *= (0.8)(0.1). The ABH procedure shows very similar behavior to the results of the BH but is closer to the nominal level because of adaptivity.

**Figure 2:**
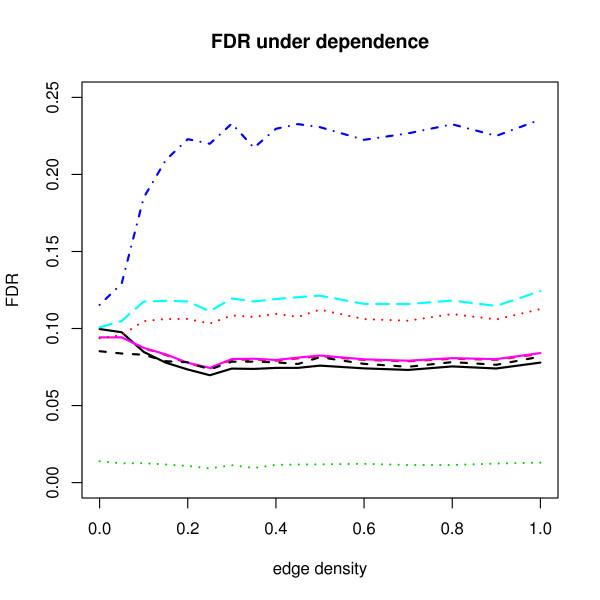
**Average FDR results under dependence when *π*_0 _= 0.95**. See Figure 1 for explanation.

Surprisingly, the point RBH estimates seem to perform better under dependence than the reference FDR. Figure 2 shows that those estimates are even close to the nominal level 0.1 while the upper limit RBH estimates in both Figures remain conservative. The difference between the two estimates is small under independence, but becomes larger as the edge density increases. The reason behind these phenomena is hard to explain because the implementation of FDR-AME is modified from the algorithms of Yekutieli et al. [12]. But, we may infer the following two points. First, as in Yekutieli et al. [12], both estimators are assumed to be less than or equal to the true FDR under the complete null hypothesis with the assumption of independence of the number of false discoveries, *V *and the number of true discoveries, *S *and the subset pivotality condition, which can be easily violated in our setting. Second, more importantly, the two estimators of $\hat{s}$(*γ*) take into account of dependence conditions differently and the $\hat{s}$(*γ*) estimator of the point RBH procedure is downward biased as explained in [12] so that the resampled FDR is estimated upward. In both Figure 1 and Figure 2, the BY procedure shows too conservative results because when *m *= 1000, $\sum_{i=1}^{1000}i^{-1}\approx 7.5$, which causes the BY adjusted *p*-values to be larger than BH adjusted *p*-values by a factor of 7.5.

**Figure 3:**
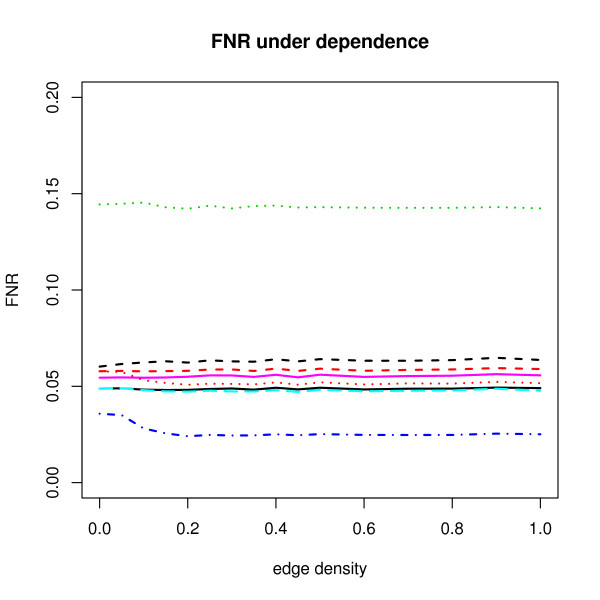
**Average FNR results under dependence when *π*_0 _= 0.8**. The *y*-axis corresponds to FNR estimates for various procedures. For the other explanation, see Figure 1.

Figure 3 shows the False Non-discovery Rate (FNR) results under dependence. The FNR is introduced by Genovese et al. [17]. It is defined as the proportion of the number of falsely accepted hypotheses over the total number of accepted hypotheses. The FNR is a dual quantity to the FDR. One may regard the FNR as a type 2 error rate if the FDR is regarded as a type 1 error in multiple testing problems. Using a single testing cut-off, we may expect that the FDR performances behave opposite to the FNR performances. Here, we observe that the BY procedure has the largest FNR. The SAM procedure has the smallest FNR while the BY procedure is most conservative and the SAM procedure is most liberal in the FDR control under dependence.

It is hard to decide that which one is recommended in practice when apparent dependence is observed. However, in this simulation, if most weight is given on adhering strict control level and gaining more power is a secondary goal, the ABH seems to be most robust and desirable under dependence cases.

**Figure 4:**
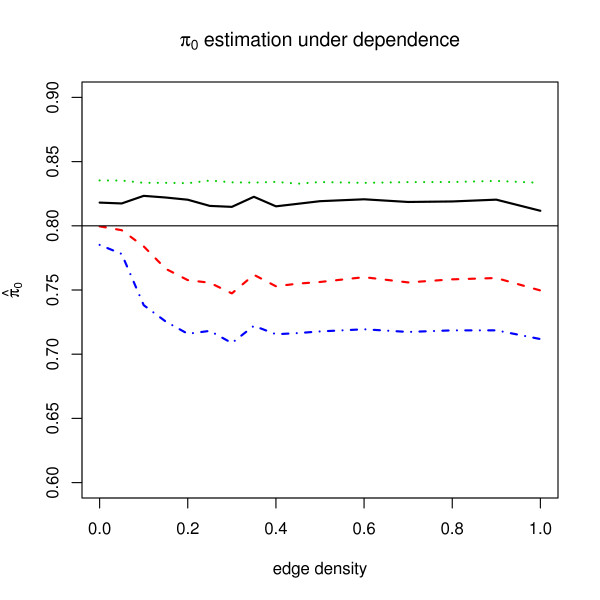
**Average *π*_0 _estimates under dependence when *π*_0 _= 0.8**. The *x*-axis corresponds to the proportion of edges in the network and the *y*-axis corresponds to *π*_0 _estimates for various procedures. The *π*_0 _estimators and corresponding lines are SAM (solid black), Qvalue (dashed red), ABH (dotted green) and the convex estimator of Langaas et al [10] (dot-dashed).

Figure 4 shows the *π*_0 _estimates for four different methods. Internal *π*_0 _estimation methods of SAM and ABH do not seem to be affected by dependence. On the contrary, *π*_0 _estimations of Qvalue and "convest" show severe sensitivity to dependency along the edge density. The latter may be improved by restricting the *p*-values density to the convex domain [10]. Interestingly, note that *π*_0 _estimations of SAM and Qvalue are based on Storey [18] and Storey et al. [19], respectively. Both of these use ${\hat{\pi }}_{0}$(*λ*) = *W*(*λ*)/((1 - *λ*)*m*) where *λ *is an intermediate parameter to compute estimates of *π*_0 _and *W*(*λ*) is the number of hypotheses whose *p*-values are greater than *λ*. In SAM, *λ *is set to 0.5 and estimates of *π*_0 _are computed while in the default method of Qvalue, the function ${\hat{\pi }}_{0}$(*λ*) of *λ *is smoothed by spline functions of order 3 on *λ *= 0, 0.01, 0.02,...,0.95.

**Figure 5:**
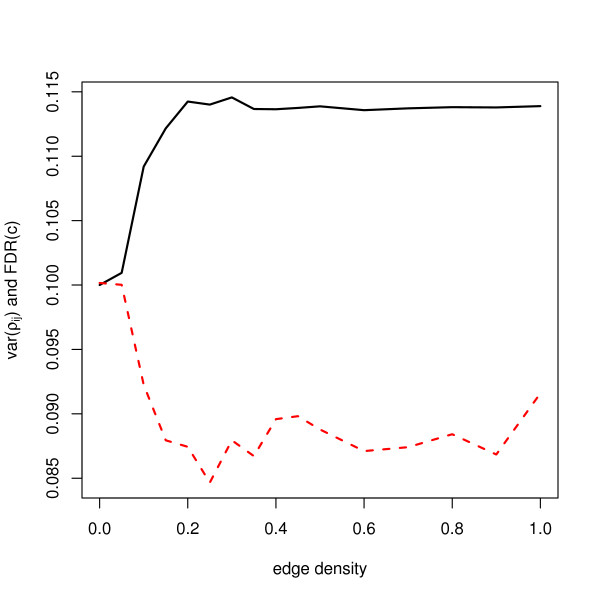
**Variances of correlations and FDR(*c*) when *π*_0 _= 0.8**. The solid line represents variance of correlations and the dashed line represents FDR(*c*). For comparison, we transform *var*(*ρ*_*ij*_) to *var*(*ρ*_*ij*_)/10 + 0.1 so that two quantities have same scale.

Besides the edge density, the strength of the present correlations also influences FDR. The variance of pairwise correlations was previously described as an important parameter in FDR estimation [20]. We show that our parameter *M*, the number of rows of the initial *Z *matrix, may be used to control it, which is suggested by the asymptotic relation, as given in equation (4). Figure 5 shows the relation between variance of correlations and FDR(*c*_*γ*_) for *M *= 1001. Up to around 0.2 of edge density, variance of correlations and FDR(*c*_0.1_) behave exactly opposite and then both quantities flatten.

**Figure 6:**
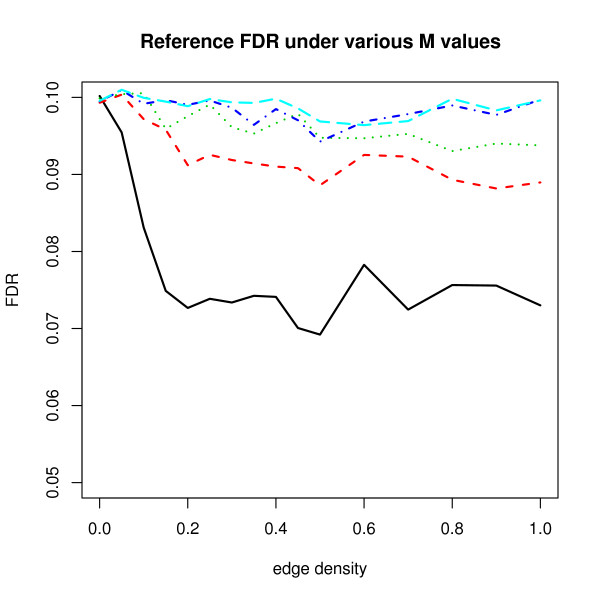
**FDR(*c*) with different *M *values**. For various *M *- *m *values, FDR(*c*) is computed. The *M *- *m *values and corresponding lines are 1001 (solid black), 1010 (dashed red), 1025 (dotted green), 1046 (dot-dashed blue) and 1073 (dashed cyan).

In Figure 6, we compare the effect of five different *M *values, 1001, 1010, 1025, 1046 and 1073 on FDR results (the reference FDR in (5)). Using (4), approximate standard deviations of correlations *ρ*_*ij *_for the five *M *values are 1/$\sqrt{3}$, 1/(2$\sqrt{3}$), 1/(3$\sqrt{3}$), 1/(4$\sqrt{3}$) and 1/(5$\sqrt{3}$). We observe that the FDR results for small *M *are more variable than that for large *M*. From (4), we expect variability almost disappears as *M *- *m *becomes large.

### An illustration with real data

In this section, we address an example on how to apply biological information such as pathways using random correlation matrices. Basically, we use estimated marginal mean and variance from data and apply pathway information such as gene sets to correlation structures. Algorithm 3 shows the detailed procedure. It uses Algorithms 1 and 2, which are discussed in the Methods section.

Algorithm 3. Application to random correlation structures to real two sample data.

1. Compute *m*-dimensional sample mean and sample variance vectors, ${\hat{\mu }}_{X},{\hat{\mu }}_{Y},{\hat{\sigma }}_{X}^{2},{\hat{\sigma }}_{Y}^{2}$ from data $X_{1},...,X_{n_{1}}$ and $Y_{1},...,Y_{n_{2}}$.

2. Prepare interested gene sets *GS*_1_,..., *GS*_*k *_and make a sequence of nested gene sets *N*_1_,..., *N*_*k *_by iterative merging. That is, for each *j *= 1,..., *k*, *N*_*j *_= *GS*_1 _∪ ... ∪ *GS*_*j*_.

3. Generate a sequence of binary adjacency matrices *A*_1_,..., *A*_*k *_from *N*_1_,..., *N*_*k*_. Components of adjacency matrices are encoded as 1 for presence of edge and 0 for absence of edge. For example, [*A*_*l*_]_*i*, *j *_= 1 means both *i*-th and *j*-th gene are in *N*_*l*_.

4. According to *A*_1_,..., *A*_*k*_, generate a sequence of random correlation matrices, *R*_1_,..., *R*_*k*_, using Algorithms 1 and 2.

5. Generate sample from $X_{1}^{*b},...,X_{n_{1}}^{*b}\sim N({\hat{\mu }}_{X},\text{diag}({\hat{\sigma }}_{X})R_{j}\text{diag}({\hat{\sigma }}_{X}))$ and $Y_{1}^{*b},...,Y_{n_{1}}^{*b}\sim N({\hat{\mu }}_{Y},\text{diag}({\hat{\sigma }}_{Y})R_{j}\text{diag}({\hat{\sigma }}_{Y}))$ for *b *= 1,..., *B*.

6. Do multiple testing *B *times and estimate average FDR from (5).

We applied the above algorithm to the "Two Class" example data of Excel add-in of SAM which consists of 1000 genes with 10 control and 10 treatment experiments. Along with the data provided, we used gene sets file, "c2.v2.symbols.gmt" for pathway information from MSigDB [21]. There are 1687 gene sets in the file and we chose those 10 gene sets (Gene Set 291, 698, 861, 885, 1069, 1177, 1179, 1237, 1345, 1453) which overlap more than 50 genes with the gene list of the "Two Class" data.

For *B *= 1000, we applied the BH FDR method with significance level 0.1 to find differentially expressed genes for each random correlation matrices. The number of detected genes and the gene lists had few variation. The median number of detected genes decreases as the edge density increases and around 100 genes were always detected regardless of the edge density, see Table 1.

**Table 1:** Median number of detected genes under increasing edge densities and the corresponding correlation matrices

	*R*_1_	*R*_2_	*R*_3_	*R*_4_	*R*_5_	*R*_6_	*R*_7_	*R*_8_	*R*_9_	*R*_10_
edge density	0.003	0.012	0.022	0.037	0.067	0.089	0.107	0.140	0.169	0.182
#total discoveries	110	110	110	110	110	109	108	107	107	106

We illustrated the different 16 genes and significance for 10 correlation structures in Table 2. In Table 2, rows represent genes and columns represent correlation matrices. The table is read as for example, ranks of frequencies of significance declaration for SSR1 were less than median detected number 110 for *R*_1_,..., *R*_5_, 108 for *R*_7 _and 107 for *R*_8_, *R*_9_.

**Table 2:** 16 genes showing different significance feature under nested 10 correlation matrices

	*R*_1_	*R*_2_	*R*_3_	*R*_4_	*R*_5_	*R*_6_	*R*_7_	*R*_8_	*R*_9_	*R*_10_
PRKCZ	1	1	1	1	1	1	1	0	1	1
HSPA4	1	1	0	0	0	1	0	0	0	1
SIAT7B	0	0	1	1	0	0	0	0	1	0
40222_s_at	1	1	1	1	1	0	1	0	1	1
36374_at	0	0	0	0	0	0	0	1	1	0
1627_at	0	0	0	0	0	1	0	0	0	0
SSR1	1	1	1	1	1	0	1	1	1	0
SEDLP	1	1	1	1	1	0	1	0	0	0
VG5Q	0	0	0	0	1	1	0	0	0	0
MAN2B1	1	1	1	1	1	1	1	1	0	1
NDUFS1	0	0	0	1	1	0	0	0	0	0
AMT	1	1	1	0	1	1	0	1	1	1
STX3A	1	1	1	1	1	1	1	1	0	1
AP3S2	1	1	1	1	1	1	1	1	0	0
SLC35A2	0	0	0	0	1	0	0	0	0	0
METTL3	1	1	1	1	0	1	1	1	1	0

Interpretation on the results of Table 2 depends on the specific correlation structures given in *R*_1_,..., *R*_10 _and there does not seem clear trends in rejections for 16 genes. Since marginal distributions of single genes do not change when we apply various correlation structures to correlation matrices of the multivariate normal distribution, the result that almost all detected genes were the same confirms our expectation.

## Discussion and Conclusion

We considered effects of dependence on FDR multiple testing results using multivariate normal samples. We found that in all our simulations, the simple adaptive Benjamini-Hochberg procedure [8] is most optimal under dependence, since it achieves relatively high power while remaining conservative. By definition, FDR is the expected value of a nonlinear function of indicator random variables of rejection. Hence, for computations of FDR, we need to take into account of the joint distribution of the indicator random variables. To focus on joint distributional properties of FDR, we have designed to observe variations of FDR in terms of variations of correlation structures and we have fixed other parameters such as marginal distributions and probabilities of rejections for true null and false null hypotheses. Therefore, our results could be additional useful guideline to FDR estimation methods which have been developed based on marginal distributional assumptions.

Nowadays, explaining high-dimensional data with conditional independence structures is quite active especially in microarray data analysis [1,22-24]. Such methods focus on testing on partial correlation coefficients. The necessary and sufficient condition of zero partial correlation is the same as (2). The results of testings on partial correlations is a network which can be used directly in our simulation framework when, for example, testing on difference of means between two groups of samples. Then, our simulation set-up can be regarded as a data-guided simulation to study whether a particular multiple testing method is useful for the data at hand. As a data-guided simulation using known gene sets [21,25], we introduce an algorithm for using real data in the Results section. Although a very slight downward trend for the number of discoveries with respect to increasing edge density (dependence) is found, we observe that the BH FDR method is very robust in this setting as well.

In our simulation study, we did not categorize test statistics. Most of the FDR methods in the Results section are based on simple gene specific *t*-statistic, while SAM uses its own statistic using the fudge factor which stabilize estimates of gene-wise variances. The effects of using such modified *t*-statistic are not clear but we can reflect those effects from the viewpoint of sample sizes. As sample sizes increase, the fudge factor of SAM shows a convergence feature, although it does not improve SAM's anti-conservative bias under dependence conditions. As an alternative to the fudge factor, the random variance model (RVM) by [26] can be used and simple replacement of the pooled variance of *t*-statistic by the RVM variance results in close control of the FDR to the nominal level under dependence in moderate to large sample size conditions. For the effects of various sample sizes on the fudge factor and *π*_0 _estimates of SAM and the RVM FDR, see Figure S1-S4 of Additional file 1.

Effect size may be another important factor in evaluating FDR methods. We consider the cases for multiple small effect sizes or very small proportion of fixed effect size, for example *π*_0 _= 0.99. In both cases, we observe overall similar patterns of the FDR estimates shown in the Results section [see Figure S8-S11 of the Additional file 1].

Generally in high-dimensional situation, we doubt that the permutational based approach to estimate joint distributional properties of test statistics always give a correct answer. In a further simulation study, the estimated spread of ordered SAM statistics under permutational null hypothesis shows to be narrower than that of the true distribution. Note that the difference becomes wider as edge density increases. This seems to cause the anti-conservative feature of SAM under dependence. For more detail on the effect of sample size and the performance of SAM and RVM, see Appendix 2 of Additional file 1.

Efron [20] notices that variance of pairwise correlations plays an important role in characterizing FDR, defined somewhat differently as the expected number of false rejections over the observed number of rejections, *E*(*V*)/*R*. We confirm this finding, but in our network-based simulation set-up, we found it natural to characterize FDR using two parameters: first, edge density to decide the proportion of interactions present and second the variance of pairwise correlations. This allows to study multiple testing methods for a given prior network.

Other interesting works on the effects of dependence on the number of false discoveries rather than FDR are Owen [27] and Korn et al. [15] who discuss that large positive correlations may result in high variation on the number of false discoveries. Under simple correlation structures, Qiu et al. [14] investigate the vulnerability of application of empirical Bayes theory under strong correlations.

One can extend our simulation framework by considering the distribution of the *Z *matrix. Until now, we have considered the constrained random correlation matrices depending on the fixed single *Z *matrix and given nested structures. Taking into account the distributional properties of *Z *as a prior, one may attain explicit posterior distribution of covariance matrices Σ_1_,...,Σ_*d*_. A family of covariance matrices as a Gaussian ensemble can also be considered as described in [28]. However, both approaches require very complicated mathematical computations so we remain these as future works.

Our simulation set-up is also useful for testing a potentially new method on *π*_0 _or FDR estimation in a dependency context. One may not have designed the procedure for the multivariate normal setting in particular; however, it seems reasonable that the new method should perform well under these conditions to be of general use. Or one may at least sketch the boundaries of the usefulness of the method in terms of type of network, edge density, and correlation strength.

## Methods

In this section, firstly, we introduce the property of conditional independence in multivariate normal distributions and its implications as graphical representations. Secondly, we introduce how to incorporate conditional independence structures to random correlation matrices and how to generate constrained random correlation matrices in a 'nested' way. Thirdly, we introduce FDR methods and *π*_0 _estimation methods used in this simulation study.

### Conditional independence correlation structures

In multivariate normal distributions, conditional independence among variables is a well established property (see chapter 5, p.129 in [29]). It states: if *X *= (*X*_1_,..., *X*_*m*_)^*T *^is a multivariate normal vector with variance-covariance matrix Σ, then

(2) $$\begin{array}{cc} X_{i}\;╨\;X_{j}|\{\text{the rest variables}\} & \text{if and only if }{\left[\Sigma^{-1}\right]}_{ij}=0. \end{array}$$

Here, "╨" represents independence between random variables.

**Figure 7:**
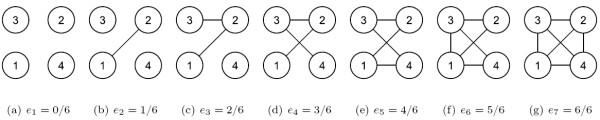
**Graphical representation of conditional independence structures when *m *= 4**. A sequence of possible nested structure is depicted when the number of nodes is 4. The left most graph represents complete independence between variables and the right most graph represents complete dependence between variables. The dependence structure of every left graph is contained to the structure of the graph right to it.

Also, the conditional independence property has a nice graphical interpretation [30]. Every node in the graph denotes a random variable and every missing edge between two nodes means that the two random variables satisfy the condition (2). If there is no edge in the graph, it corresponds to independence structure, that is, the corresponding variance-covariance matrix is the identity matrix. If nodes are fully connected, we may regard it as completely dependent structure. For *m *= 4, we illustrate a sequence of graphs with various conditional independence structures in Figure 7.

Given *m *dimensional multivariate normal distribution, however, there are $2^{\left(\begin{array}{c} m\\ 2 \end{array}\right)}$ different conditional independence structures or graphs. Comparing every pair of structures for large *m *is infeasible. But note that the class of structures is a partially ordered set by inclusions or exclusions of edges. In the partially ordered set, the minimal element is the totally independence structure corresponding to identity variance-covariance matrix in a matrix form. Maximal elements are completely dependent structures without any conditional independence constraints, that is every entry of inverse of the variance-covariance matrix is non-zero. Hence, it is meaningful to regard a partially ordered path as a sequence of dependence conditions as in the single correlation structures. Comparisons through a partially ordered path give insights on dependence effects. Then, it is natural to regard the proportions of edges in a path as a dependence parameter. Figure 7 shows such an instance of the partially ordered path. In following sections, we use the term 'edge density' as proportion of edges and by a 'nested' sequence we mean a partially ordered path of conditional independence structures.

### Generating constrained random correlation matrices

Unconstrained random correlation matrices are generated simply by products of matrix transposes and its standardizations [31]. Let *Z *be an *M *× *m *matrix whose entries are generated from independent standard normal distributions. If *M *is greater than *m*, then the matrix *W *= (*Z*^*T*^*Z*)^-1 ^is a symmetric positive definite matrix with probability one. *M *will be used as a parameter to control the strength of the correlations. After standardizing *W*, we obtain

(3) Σ = diag(*W*)^-1/2 ^*W *diag(*W*)^-1/2^.

Then Σ is an unconstrained random positive definite correlation matrix.

To incorporate conditional independence structures into the process (3), we need to transform the *Z *matrix into a matrix $\tilde{Z}$ such that $\tilde{Z}$ bears the information on the structures. These transformations are basically based on successive orthogonal projections. For a simple example, consider imposing the simple constraint *X*_1 _╨*X*_2_|{rest} on Σ in (3). We carry out the following steps. First, we generate the *Z *= [*z*_1_,..., *z*_*m*_] matrix with *m *column vectors. Second, we let ${\tilde{z}}_{2}=z_{2}-z_{1}{\left(z_{1}^{T}z_{1}\right)}^{-1}z_{1}^{T}z_{2}$, then ${\tilde{z}}_{2}$ is the residual vector of *z*_2 _projected onto the linear space spanned by *z*_1_. Finally, if we replace matrix *W *in (3) by ${\left({\tilde{Z}}^{T}\tilde{Z}\right)}^{-1}$ where $\tilde{Z}=[z_{1},{\tilde{z}}_{2},z_{3},...,z_{m}]$, then Σ is a random correlation matrix satisfying the constraint [Σ^-1^]_12 _= 0 by construction.

For imposing a large number of conditional independence constraints, we provide general steps below. First, we introduce a constraint matrix *J*. *J *is an *m *× *m *symmetric binary matrix whose diagonal entries are one. Its off-diagonal entries equal to zero represent conditional independence between the row and column variables. These also correspond to the missing edges in the graph. So, the *J *matrix is useful in the sense that it directly shows its whole structures and it gives computational convenience when one considers generating random structures. For the above example, the (1, 2) and (2, 1) positions of the *J *matrix are set [*J*]_12 _= [*J*]_21 _= 0 and [*J*]_*ij *_= 1 for the other entries. Table 3 shows the constraint matrices according to the conditional independence structures of Figure 7.

**Table 3:** Constraint matrices corresponding to the graphs in Figure 7

(a) *e*_1 _= 0/6 $\left[\begin{array}{cccc} 1 & 0 & 0 & 0\\ & 1 & 0 & 0\\ & & 1 & 0\\ & & & 1 \end{array}\right]$	(b) *e*_2 _= 1/6 $\left[\begin{array}{cccc} 1 & 1 & 0 & 0\\ & 1 & 0 & 0\\ & & 1 & 0\\ & & & 1 \end{array}\right]$	(c) *e*_3 _= 2/6 $\left[\begin{array}{cccc} 1 & 1 & 0 & 0\\ & 1 & 1 & 0\\ & & 1 & 0\\ & & & 1 \end{array}\right]$	(d) *e*_4 _= 3/6 $\left[\begin{array}{cccc} 1 & 1 & 0 & 0\\ & 1 & 1 & 0\\ & & 1 & 1\\ & & & 1 \end{array}\right]$
(e) *e*_5 _= 4/6 $\left[\begin{array}{cccc} 1 & 1 & 0 & 1\\ & 1 & 1 & 0\\ & & 1 & 1\\ & & & 1 \end{array}\right]$	(f) *e*_6 _= 5/6 $\left[\begin{array}{cccc} 1 & 1 & 1 & 1\\ & 1 & 1 & 0\\ & & 1 & 1\\ & & & 1 \end{array}\right]$	(g) *e*_7 _= 6/6 $\left[\begin{array}{cccc} 1 & 1 & 1 & 1\\ & 1 & 1 & 1\\ & & 1 & 1\\ & & & 1 \end{array}\right]$	

Now, we provide two algorithms used for our simulation studies. Basically, we apply the second algorithm and the first one is included in the second one.

Algorithm 1. Generating a constrained random correlation matrices given constraint matrix *J*.

1. Generate an *Z *= [*z*_1_,..., *z*_*m*_] matrix from standard normal distributions.

2. Let *I*_*l *_= {*k *: [*J*]_*kl *_= 0 for *k *= 0,..., *l *- 1} for *l *= 1,..., *m *and $z_{I_{l}}$ be the matrix consisting of column vectors of *Z *with indices in *I*_*l*_.

3. Let ${\tilde{z}}_{1}$ = *z*_1_.

4. For each *l *= 2,..., *m*, ${\tilde{z}}_{l}$ = *z*_*l *_- *P*_*l*_*z*_*l *_where $P_{l}={\tilde{z}}_{I_{l}}{\left({\tilde{z}}_{I_{l}}^{T}{\tilde{z}}_{I_{l}}\right)}^{-1}{\tilde{z}}_{I_{l}}^{T}$, i.e. the projection of *z*_*l *_onto the space spanned by {${\tilde{z}}_{i}$ : *i *∈ *I*_*l*_}.

5. Let $\tilde{Z}=[{\tilde{z}}_{1},...,{\tilde{z}}_{m}]$. Then Σ with $W={\left({\tilde{Z}}^{T}\tilde{Z}\right)}^{-1}$ is a random correlation matrix with constraint matrix *J*.

Algorithm 2. Generating a nested sequence of constrained random correlation matrices.

1. Generate a *Z *matrix from standard normal distributions.

2. Generate a sequence of edge densities, *e*_1_,..., *e*_*d *_with 0 = *e*_1 _< ⋯ <*e*_*d *_= 1.

3. Generate a nested sequence of random constraint matrices *J*_1_,..., *J*_*d *_according to edge densities, *e*_1_,..., *e*_*d*_. Note the proportion of non-zero off-diagonal elements in *J*_*i *_is *e*_*i*_.

4. Given the *Z *matrix, generate Σ_1_,...,Σ_*d *_according constraint matrices *J*_1_,..., *J*_*d *_by Algorithm 1. Then Σ_1 _= *I *and the sequence of random correlation matrices has nested conditional independence structures.

### Variance of correlations and the choice of *M*

In this simulation study we assume that dependence conditions are determined by conditional independence structures of random correlation matrices. However, it is meaningful to understand the relation between structural dependence and dependence given by pairwise correlations. Even though the randomness in the generation process (3) makes it difficult to grasp the relation, average variance of pairwise correlations depends on the parameter *M*, which is the number of rows of the initial *Z *matrix. The role of the parameter *M *used in generating the *Z *matrix is to control the variance of pairwise correlations, which on its turn is an important parameter in FDR estimation [20]. The expectation and variance of pairwise correlations *ρ*_*ij *_are approximately

(4) $$\begin{array}{cc} E(\rho_{ij})=O({\left(M-m+2\right)}^{-2}), & var(\rho_{ij})=\frac{1}{M-m+2}+O({\left(M-m+2\right)}^{-2}) \end{array}$$

when *Z *is generated from standard normal distributions [see Appendix 1 of Additional file 1]. Hence for large *M*, we expect average pairwise correlations are close to zero and the effect of dependence when *M *is large is almost ignorable.

Average variances of off-diagonal entries in (3) decrease very quickly to zero as *M *increases. Hence, in this paper, when *m *= 1000, we focus on FDR results for *M *= 1001 since this value illustrates the effects of dependence in the most unrestricted way. For large *M *- *m*, variances of pairwise correlations are close to zero and the effects are almost negligible. In Figure 6, we show the FDR results for such a case.

### Simulation details

We perform unpaired two group *t*-test under multivariate normal distribution. Each group has the same correlation matrix, but a proportion *π*_0 _of the total number of hypotheses has different mean. The mean difference is computed given fixed probabilities of rejection of true and false null hypotheses. General simulation steps are the followings.

1. Find *c*_*γ *_satisfying FDR(*c*_*γ*_) = *γ *under independence assumption.

2. Generate random correlation matrices Σ_1_,..., *Σ*_*d *_from given structures in Algorithm 2.

3. For each Σ_*j*_, $X_{1},...,X_{n_{1}}$ ~ *N*_*m*_(*μ*_*X*_, Σ_*j*_) and $Y_{1},...,Y_{n_{2}}$ ~ *N*_*m*_(*μ*_*Y*_, Σ_*j*_).

4. Apply various multiple testing procedures to these data and compare the corresponding results of FDR, FNR and *π*_0 _estimates.

In this simulation study, we also intend to observe generic features of FDR behavior under dependence circumstances. Therefore, we consider a reference FDR. It is hard to find testing cut-offs which produce exact control under dependence conditions. Hence under the independence condition and significance level *γ*, we compute a testing cut-off *c*_*γ *_such that FDR(*c*_*γ*_) = *γ *[7] and we apply this cut-off to dependence cases. Using a Monte-Carlo method, we obtain approximate FDR values for fixed cut-off *c*_*γ *_under dependence conditions. Hence from *B *random samples, we compute the following quantity for each *i *= 1,..., *d*,

(5) $$\text{FDR}(c_{\gamma },\Sigma_{i})\approx \frac{1}{B}\sum_{b=1}^{B}\frac{v_{b,i}}{v_{b,i}+s_{b,i}}.$$

### FDR procedures, *π*_0 _estimation methods and software used in the simulations

We briefly introduce the FDR implementations used in the simulation studies. Most of them are regularly used and all of them are developed in R software packages [32].

• Benjamini-Hochberg procedure (BH): Implemented FDR control by a linear step-up procedure [2]. From ordered observed *p*-values *p*_(1)_,..., *p*_(*m*)_, it finds maximal *k *such that $p_{\left(k\right)}\leq \gamma \frac{k}{m}$ given significance level *γ*. It is known to control FDR at level $\gamma \frac{m_{0}}{m}$ under independence assumption of test statistics. *π*_0 _estimation procedure is not implemented, hence *π*_0 _is assumed to be 1. We use R package *multtest *for this procedure.

• Benjamini-Yekutieli procedure (BY): Benjamini et al. [3] extends the BH procedure to control FDR at level *γ *under arbitrary dependence conditions. It uses the linear step-up procedure, and it finds maximal *k *such that $p_{\left(k\right)}\leq \gamma \frac{k}{m}{\left(\sum_{i=1}^{m}i^{-1}\right)}^{-1}$. We use R package *multtest *for this procedure.

• Adaptive Benjamini-Hochberg procedure (ABH): The ABH procedure improves the BH procedure by estimating *m*_0_. Given significance level *γ*, the two-stage ABH procedure first performs the linear step-up BH procedure to find *r*_1_, the number of rejected hypotheses at level γ* = *γ*/(1 + *γ*). It estimates ${\hat{m}}_{0}$ as *m *- *r*_1 _and then applies $\gamma^{*}\frac{m}{{\hat{m}}_{0}}$ as a new significance level in the second step. Under the independence assumption of test statistics, ABH is known to control FDR at level *γ *[8]. We use R package *FDR-AME *for this procedure.

• Significance Analysis of Microarray (SAM): Based on [6], the SAM procedure is developed. For two-class, unpaired data, it uses a *t*-statistic combined with a fudge factor which makes test statistics more stable when sample variance is very small. Using permutations and a user-specified cut-off, it produces asymmetric testing results. To apply the same significance level *γ *as other FDR procedures, we set median FDR level to be *γ *instead of using the user-specified cut-off. We use R package *samr *with internal permutation number 200.

• Qvalue: Storey [18] proposes a new multiple testing criterion *q*-value based on pFDR. pFDR is defined as the expected proportion of the number of false rejections over the number of rejections given the number of rejections is at least one. *q*-value is the minimum pFDR where the statistic is declared significant. The estimates of *q*-values are computed from a function of individual *p*-values while preserving the order of *p*-values. We use R package *qvalue *and choose the default option "smoother" as "pi0.method".

• Resampling based FDR adjustments (RBH): Resampling based FDR estimation is based on the resampling distribution of *p*-values under the complete null hypothesis. Basically, it is defined as *E*_*R*(*γ*)* _[*R*(*γ*)*/(*R*(*γ*)* + $\hat{s}$(*γ*))] where R*(*γ*) is the number of resampling-based *p*-values less than *γ*. Two estimators conditioned on $\hat{s}$(*γ*) are proposed. The point RBH estimator is based on $\hat{s}$(*γ*) = *r*(*γ*) - *mγ *and the upper limit RBH estimator is based on $\hat{s}(\gamma )=r(\gamma )-r_{\beta }^{*}(\gamma )$ where $r_{\beta }^{*}(\gamma )$ is 1 - *β *quantile of R*(*γ*) [12]. We use R package *FDR-AME *for this procedure.

ABH, SAM and Qvalue contain internal *π*_0 _estimation. Recently, another *π*_0 _estimation method is introduced by Langaas et al. [10]. Here, *p*-values are modeled as *f*(*p*) = *π*_0 _+ (1 - *π*_0_)*h*(*p*) where *h*(*p*) is a convex decreasing density of false null hypotheses with *h*(1) = 0. In this set-up, nonparametric maximum likelihood estimation is employed to compute estimate of *π*_0_. For the case of non-convexity of *f*, the authors advise to restrict the domain to the convex part of *f*, but this is not implemented yet. We use the *convest *function in the *limma *R packages in the default option.

### Simulation program

We developed R code [32] for this simulation studies. The code can also be used in a supervised sense, using known gene sets. Please contact the authors for obtaining the R program.

## Authors' contributions

Both authors contributed to conceptual ideas of this study and writing of this article. KIK developed algorithms and implemented the R program.

## Supplementary Material

Additional file 1 Kim_VDWiel_Supp.pdf consists of Appendix 1, 2 and additional figures. Appendix 1 contains a proof for equation (4) and Appendix 2 contains an analysis for the SAM estimation of FDR under dependence. Click here for file
