# Supplementary material for: Effects of dependence in high-dimensional multiple testing problems
Source: BMC Bioinformatics. 2008 Feb 25;9:114. doi: 10.1186/1471-2105-9-114 (PMC2375137; doi:10.1186/1471-2105-9-114)
Supplement: Additional file 1 — Kim_VDWiel_Supp.pdf consists of Appendix 1, 2 and additional figures. Appendix 1 contains a proof for equation (4) and Appendix 2 contains an analysis for the SAM estimation of FDR under dependence. [file 1471-2105-9-114-S1.pdf]

Appendix 1

Expectations and variances of off-diagonal elements of Σ in equation (4) are obtained basically from the distribution of sample correlation coefficients of bivariate normal sample. We need the following three propositions.

Proposition 1 *[An application of Theorem 5.1.5, p.151, p.156-157, [2]] Let* Σ *be distributed as* $\mathcal{W}_2(n, V)$ *the Wishart distribution of degree of freedom* n *and parameter* V*. Then the expectation and the variance of* $r = \sigma_{12}/\sqrt{\sigma_{11}\sigma_{22}}$ *are*

$$E(r) = \rho - \frac{\rho(1-\rho)^2}{2n} + O(n^{-2}), \qquad var(r) = \frac{(1-\rho^2)^2}{n} + O(n^{-2})$$

where

$$\Sigma = \begin{bmatrix} \sigma_{11} & \sigma_{12} \\ \sigma_{21} & \sigma_{22} \end{bmatrix}, \qquad V = \begin{bmatrix} 1 & \rho \\ \rho & 1 \end{bmatrix}.$$

Proposition 2 *[Theorem 3.2.5, p.92, [2]] If* $\Sigma \sim \mathcal{W}_m(n, V)$ *and* C *is* $k \times m$ *of rank* k *then* $C\Sigma C^T \sim \mathcal{W}_k(n, CVC^T)$*.*

Proposition 3 *[Theorem 3.2.10 (i), p.93, [2]] Suppose* $\Sigma \sim \mathcal{W}_m(n, V)$ *where*

$$\Sigma = \begin{bmatrix} \Sigma_{11} & \Sigma_{12} \\ \Sigma_{21} & \Sigma_{22} \end{bmatrix}, \qquad V = \begin{bmatrix} V_{11} & V_{12} \\ V_{21} & V_{22} \end{bmatrix}$$

and put $\Sigma_{11\cdot 2} = \Sigma_{11} - \Sigma_{12}\Sigma_{22}^{-1}\Sigma_{21}$ *and* $V_{11\cdot 2} = V_{11} - V_{12}V_{22}^{-1}V_{21}$*. Then*

$$\Sigma_{11\cdot 2} \sim \mathcal{W}_k(n - m + k, V_{11\cdot 2})$$

where Σ_{11} *and* V_{11} *are* $k \times k$ *matrices.*

Now, let $S = Z^T Z$ and $W = S^{-1}$ where Z is an $M \times m$ matrix whose row vectors are generated from $\mathcal{N}_m(0, V)$ independently and let $W_{11} = \begin{pmatrix} w_{ii} & w_{ij} \\ w_{ji} & w_{jj} \end{pmatrix}$ for some indices i and j, then from Proposition 3 we obtain $W_{11}^{-1} = S_{11\cdot 2} \sim \mathcal{W}_2(M - m + 2, V_{11\cdot 2})$ and $U = \begin{pmatrix} u_{11} & u_{12} \\ u_{21} & u_{22} \end{pmatrix} = \mathrm{diag}(V_{11\cdot 2})^{-1/2} W_{11}^{-1} \mathrm{diag}(V_{11\cdot 2})^{-1/2} \sim \mathcal{W}_2(M - m + 2, \left(\begin{smallmatrix} 1 & \rho \\ \rho & 1 \end{smallmatrix}\right))$ for some ρ from Proposition 2. Let $R = W_{11}^{-1} = \begin{pmatrix} r_{11} & r_{12} \\ r_{21} & r_{22} \end{pmatrix}$. Then the following equations hold by simple calculations.

$$\frac{r_{12}}{\sqrt{r_{11}r_{22}}} = \frac{u_{12}}{\sqrt{u_{11}u_{22}}} = -\frac{w_{ij}}{\sqrt{w_{ii}w_{jj}}} = -\rho_{ij}$$

where ρ_{ij} is the (i,j)-th component of Σ in equation (3). Therefore, from Proposition 1,

$$E\left(\frac{u_{12}}{\sqrt{u_{11}u_{22}}}\right) = \rho - \frac{\rho(1-\rho)^2}{2(M-m+2)} + O((M-m+2)^{-2}),$$

$$var\left(\frac{u_{12}}{\sqrt{u_{11}u_{22}}}\right) = \frac{(1-\rho^2)^2}{M-m+2} + O((M-m+2)^{-2}).$$

Since $V = I_{m \times m}$, $\rho = 0$ in the simulations, we obtain

$$E(\rho_{ij}) = E(\frac{w_{ij}}{\sqrt{w_{ii}w_{jj}}}) = O((M-m+2)^{-2}),$$

$$var(\rho_{ij}) = var(\frac{w_{ij}}{\sqrt{w_{ii}w_{jj}}}) = \frac{1}{M-m+2} + O((M-m+2)^{-2}).$$

Appendix 2

Figure S1 shows results of FDR estimates of SAM under dependence. In the figure, the bias patterns of SAM still exist even for large sample size and we investigate possible reasons for that phenomena based on three distinct factors of SAM.

To understand these biases of SAM under strong dependence in estimating FDR, first we considered that SAM uses a test statistic different from the ordinary two sample t-statistic, which incorporates the idea of stabilization of gene-wise variance. Second, the π_0 estimation of SAM was considered. Third, since null distributions of the SAM statistic are estimated by permutation, we compared the true distribution and the permutational null distribution by the Monte Carlo method. Additionally, the random variance model [3] which assumes an inverse gamma distribution for overall gene variance was used to estimate FDR and was compared with SAM's gene-wise variance estimation.

We used the same correlation matrices as in the Results section for $\pi_0 = 0.8$. For the Monte Carlo study, we generated data for $B = 1000$ times. In particular, sample sizes are given $10, 25, 50$ and 100 to see effects of high dimensionality on the FDR estimation.

First, we considered the fudge factor s_0 of SAM which stabilizes gene-wise variance estimations. Figure S4 shows estimates of the fudge factor decrease as sample size increases. Hence for sufficient large sample size, we may expect the SAM statistic behaves similar to the ordinary two group t-statistic and dependence patterns for FDR estimates of SAM can be similar to those of two sample t-statistic. Variance modeling approach by [3] also shows that FDR estimates of their approach become closer to the nominal level 0.1 as sample size increases. Therefore, for large sample, the random variance model suggested by [3] performed well on overall FDR estimation while the convergence of the fudge factor of SAM estimation doesn't seem to decrease the biases of SAM FDR estimation.

Second, Figure S3 shows π_0 estimates of SAM become closer to true $\pi_0 = 0.8$ as sample size increases. Similarly, we can expect that SAM estimates of π_0 converge to true π_0 when sample size is sufficiently large. Also, the performance of π_0 estimation, likewise the fudge factor of SAM doesn't seem to improve the performance of SAM FDR estimation.

Third, we considered distributions of permutational null model implemented in SAM. Since FDR estimation mainly concerns tail distributions of test statistics, we computed average 5th and 95th quantiles under permutational null hypothesis for $B = 1000$ times. Figure S5, S6 and S7 show that the spread of

5th and 95th quantiles of true distribution of dependence case (edge density is 0.2) is wider than that of independence case (edge density is 0.0). Considering the following SAM FDR estimation equation, we may conclude that since the tail quantiles of true distribution are wider, more false positives are rejected in the numerator of the equation and that causes SAM FDR estimation to be larger than those of the SAM permutational null distribution. Comparing Figure S6 with S7, we observe that this phenomena do not disappear even when we increase the number of permutation from 200 to 1000. Therefore SAM's biases under dependence circumstance as in the Results section seem to be caused by the high-dimensionality and insufficient performance of permutation under that situation. Note SAM FDR estimation equation is

$$\widehat{\mathrm{FDR}}(\Delta) = \hat{\pi}_0 \frac{\#\{d^{*b}_{(i)} \in \Gamma(\Delta)\}/B}{\#\{d_{(i)} \in \Gamma(\Delta)\}}$$

where $\Gamma(\Delta) = [\mathrm{cut}_{low}(\Delta), \mathrm{cut}_{up}(\Delta)]^c$ and $d_i = \bar{x}_i/(s_i + s_0)$. For detailed explanation of the above equation, see the Users guide and technical document of SAM.

References

[1] Mette Langaas, Bo Henry Lindqvist, and Egil Ferkingstad. Estimating the proportion of true null hypotheses, with application to DNA microarray data. *J. R. Stat. Soc. Ser. B Stat. Methodol.*, 67(4):555–572, 2005.

[2] Robb J. Muirhead. *Aspects of multivariate statistical theory.* John Wiley & Sons Inc., New York, 1982. Wiley Series in Probability and Mathematical Statistics.

[3] GW Wright and R. Simon. A random variance model for detection of differential gene expression in small microarray experiments. *Bioinformatics*, 19:2448–55, 2003.

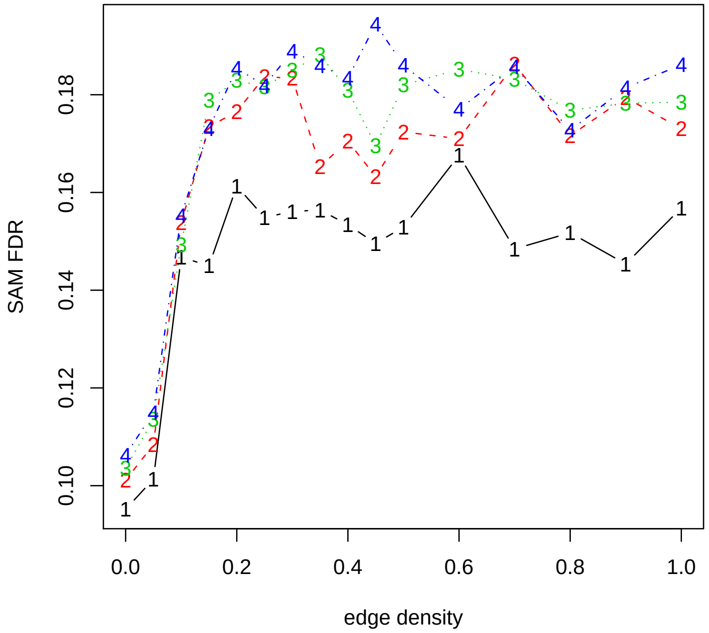

Figure S1: FDR estimation using SAM when sample size are 10 (solid 1), 25 (dashed 2), 50 (dotted 3), 100 (dash-dotted 4).

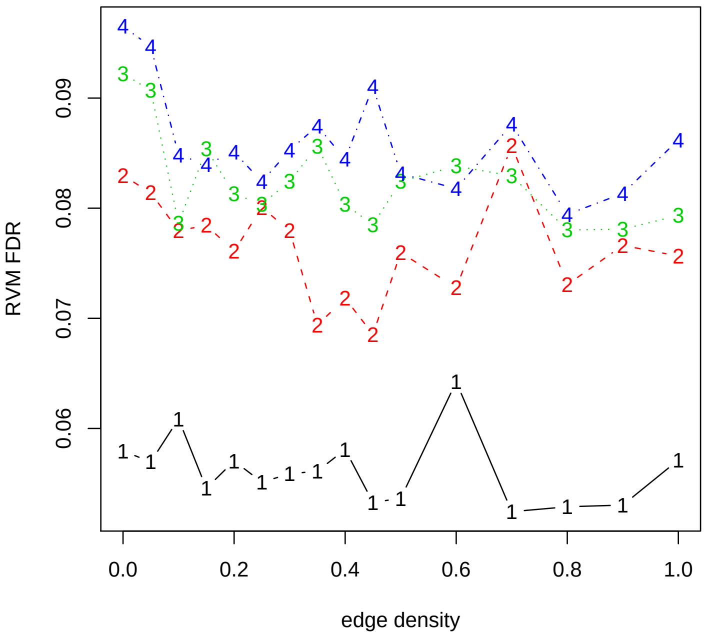

Figure S2: FDR estimation using random variance model when sample size are 10 (solid 1), 25 (dashed 2), 50 (dotted 3), 100 (dash-dotted 4).

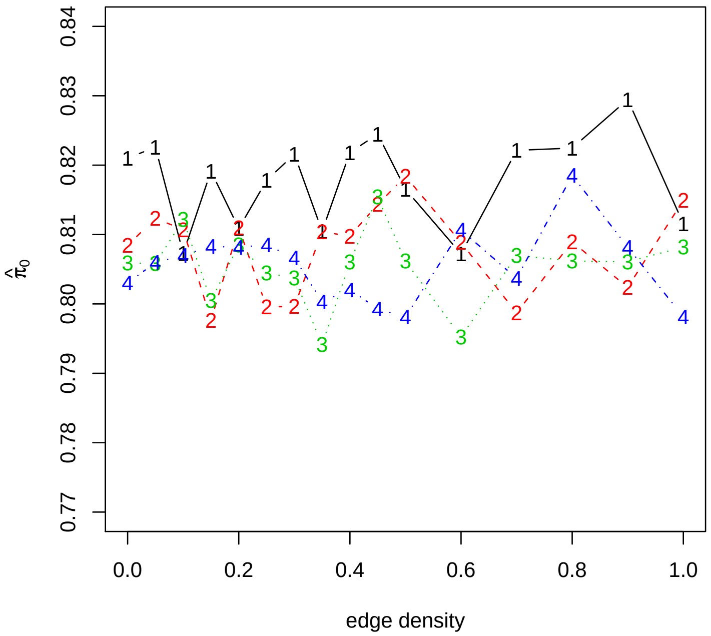

Figure S3: π_0 estimation using SAM when sample size are 10 (solid 1), 25 (dashed 2), 50 (dotted 3), 100 (dash-dotted 4).

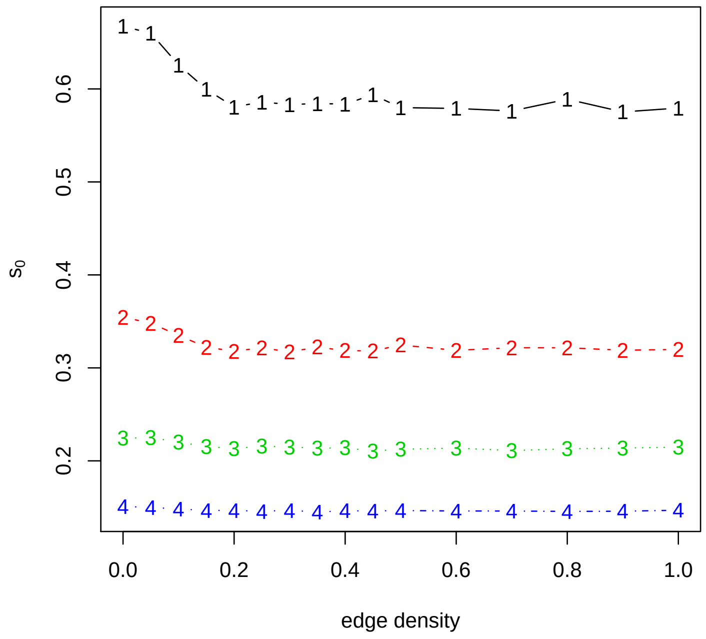

Figure S4: s_0 estimation using SAM when sample size are 10 (solid 1), 25 (dashed 2), 50 (dotted 3), 100 (dash-dotted 4).

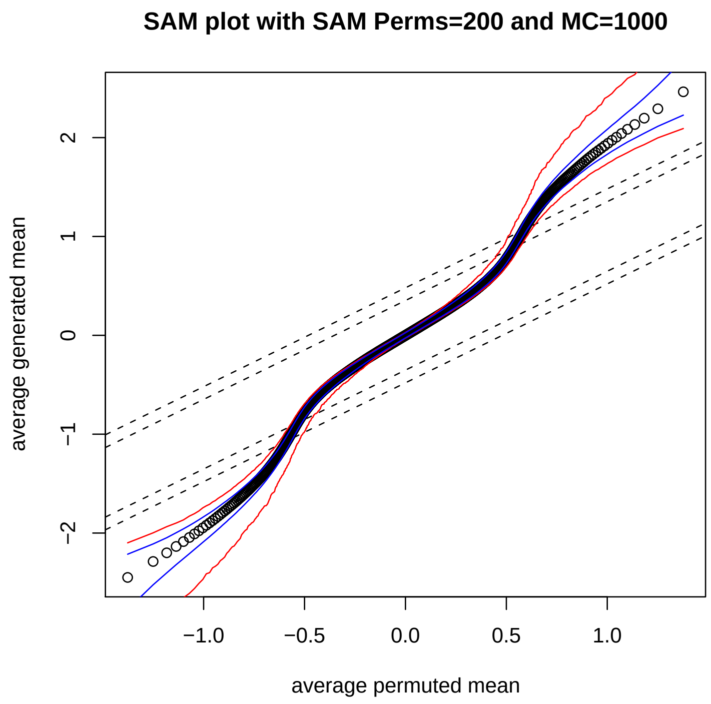

Figure S5: 5th and 95th quantiles for permutation distribution (blue line), estimated true distribution (red line) and Δ cut-offs (dashed line) for SAM when internal SAM permutation number is 200 and 1000 generated sample based on the Monte Carlo method. Edge density of this plot is 0.0.

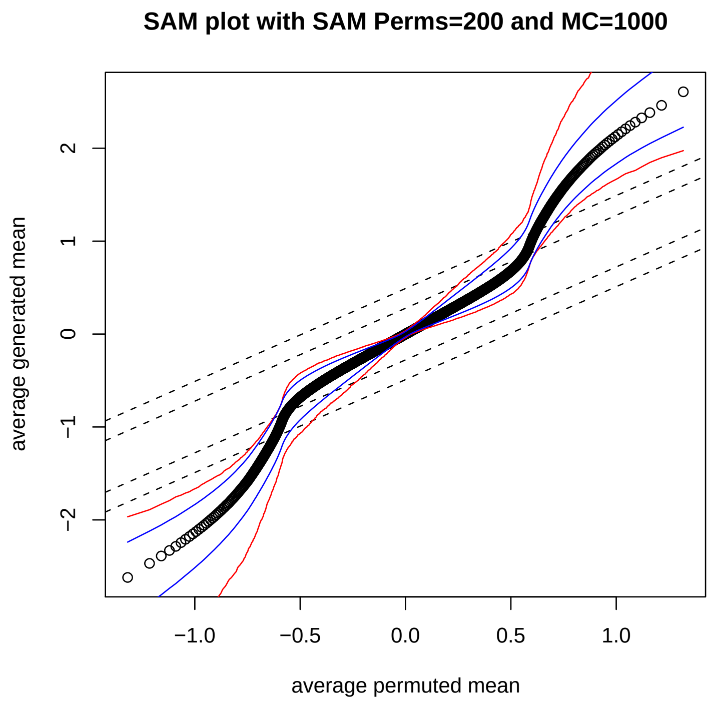

Figure S6: 5th and 95th quantiles for permutation distribution (blue line), estimated true distribution (red line) and Δ cut-offs (dashed line) for SAM when internal SAM permutation number is 200 and 1000 generated sample based on the Monte Carlo method. Edge density of this plot is 0.2.

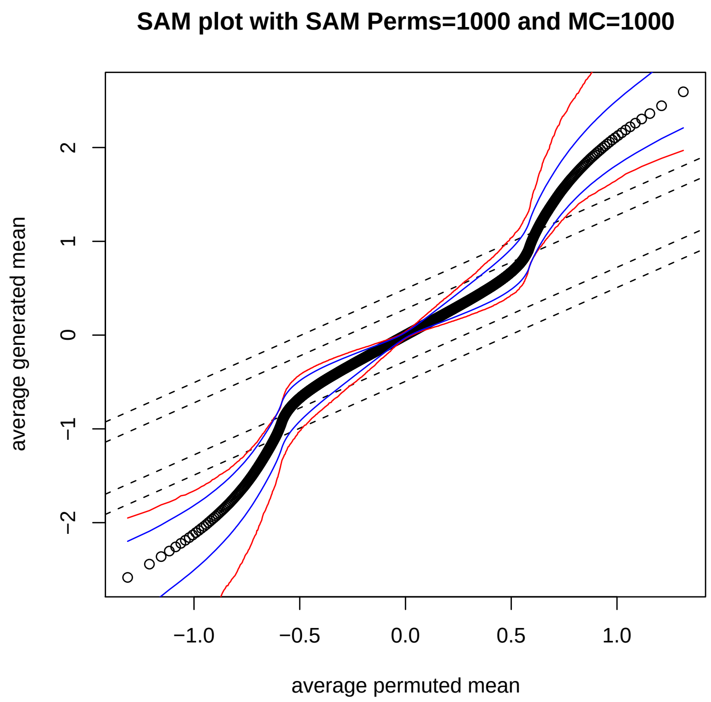

Figure S7: 5th and 95th quantiles for permutation distribution (blue line), estimated true distribution (red line) and Δ cut-offs (dashed line) for SAM when internal SAM permutation number is 1000 and 1000 generated sample based on the Monte Carlo method. Edge density of this plot is 0.2.

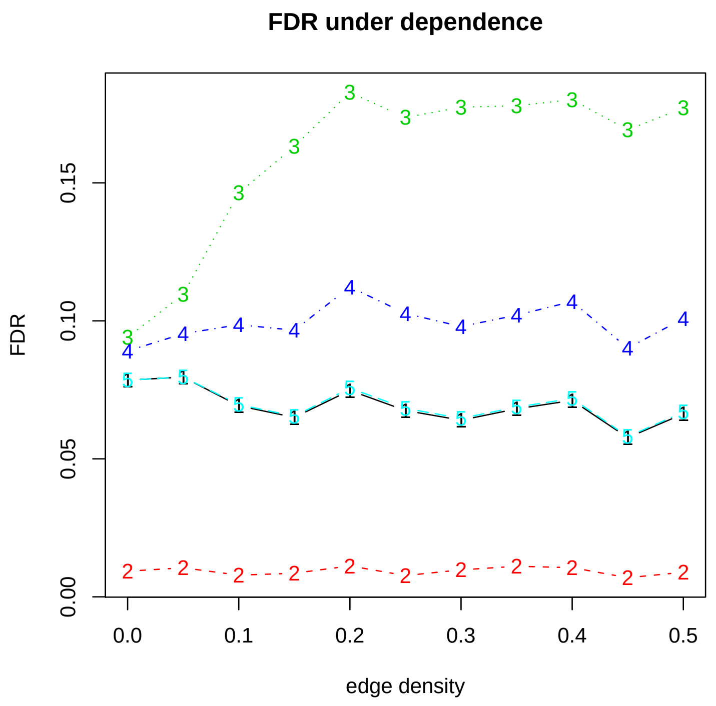

Figure S8: Average FDR results under dependence when true difference is distributed as standard normal and $\pi_0 = 0.8$, BH(1, dashed black), BY(2, dashed red), SAM(3, dotted green), Qvalue(4, dot-dashed blue), ABH(5, dashed cyan).

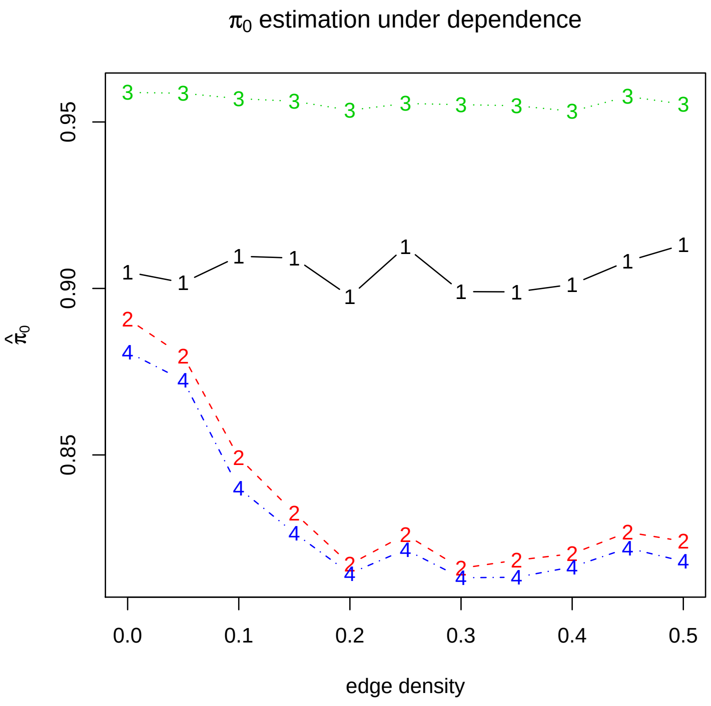

Figure S9: Average π_0 estimates under dependence when true difference is distributed as standard normal and $\pi_0 = 0.8$, SAM(1, solid black), Qvalue(2, dashed red), ABH(3, dot-dotted green), Convex(4, dotted blue).

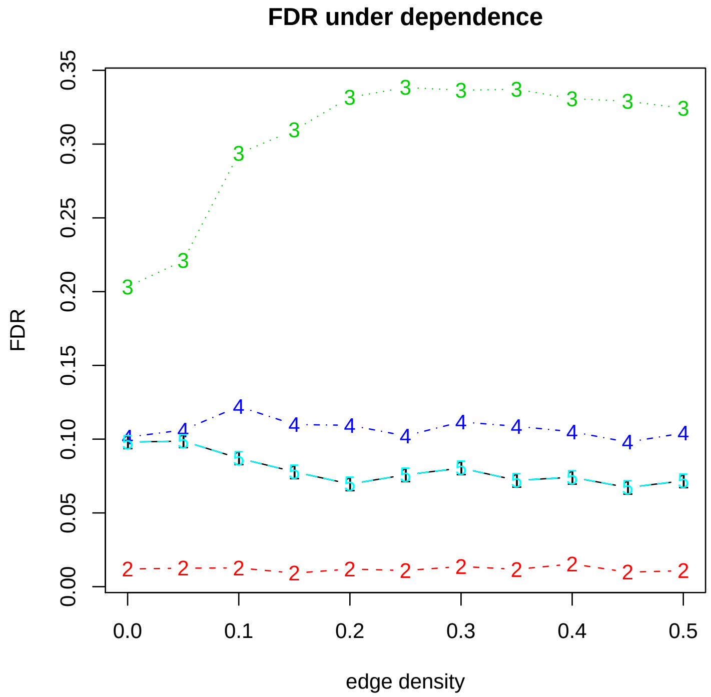

Figure S10: Average FDR results under dependence when $\pi_0 = 0.99$ and fixed true difference, BH(1, dashed black), BY(2, dashed red), SAM(3, dotted green), Qvalue(4, dot-dashed blue), ABH(5, dashed cyan).

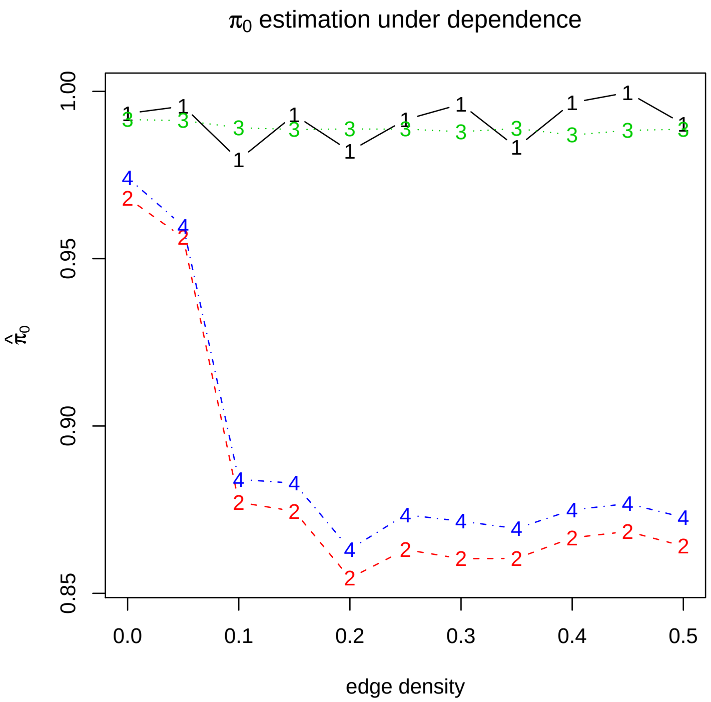

Figure S11: Average π_0 estimates under dependence when $\pi_0 = 0.99$ and fixed true difference, SAM(1, solid black), Qvalue(2, dashed red), ABH(3, dot-dotted green), Convex(4, dotted blue).

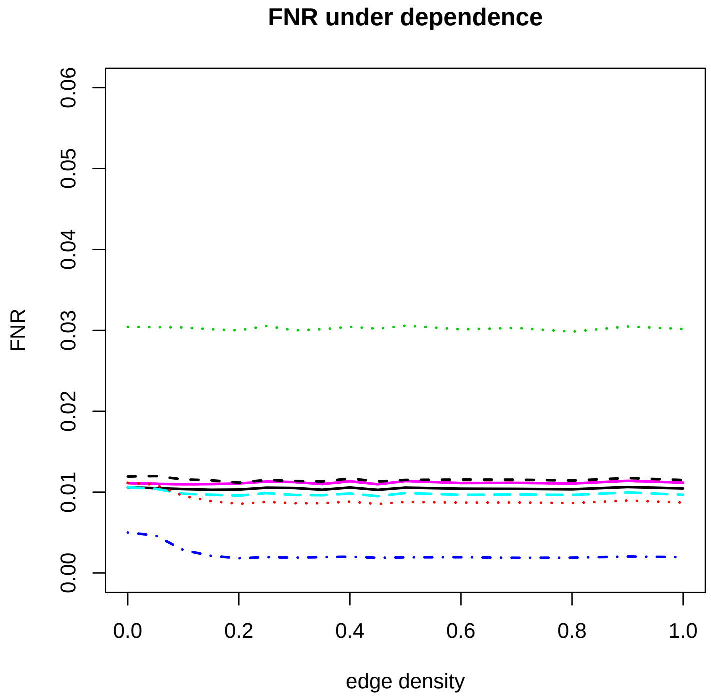

Figure S12: Average FNR results under dependence when $\pi_0 = 0.95$. BH (dashed red), BY (dotted green), SAM (dot-dashed blue), Qvalue (dashed cyan), ABH (purple), the upper limit RBH (dashed black), the point RBH (dotted red).

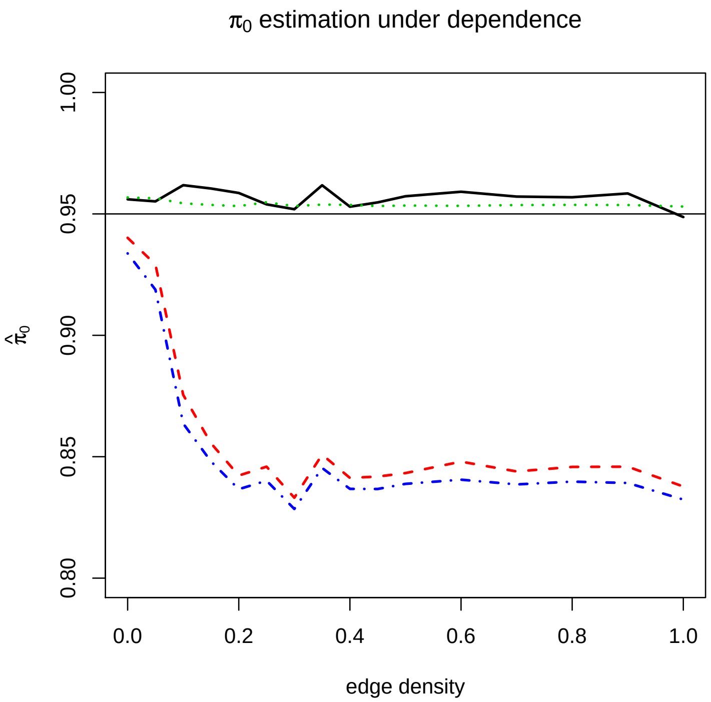

Figure S13: Average π_0 estimates under dependence when $\pi_0 = 0.95$. SAM (solid black), Qvalue (dashed red), ABH (dotted green) and the convex estimator of Langaas et al[1] (dot-dashed).

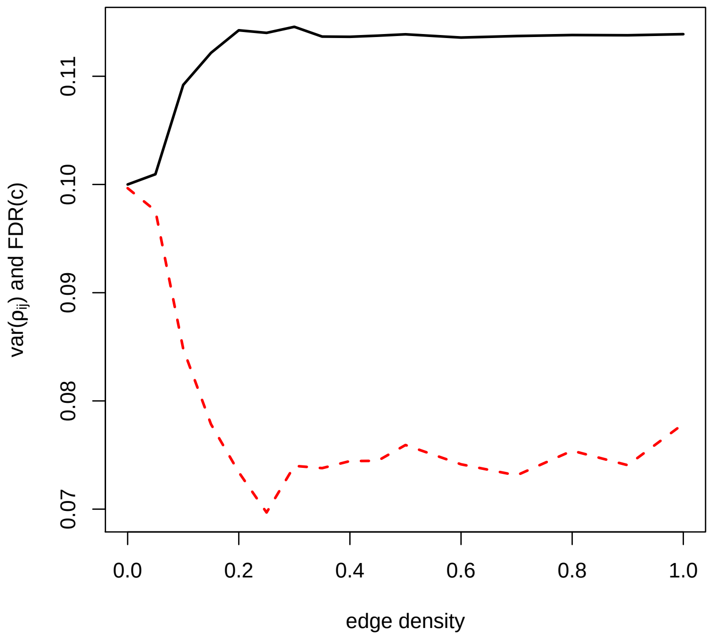

Figure S14: Variances of correlations and FDR($c_{0.1}$) when $\pi_0 = 0.95$. The solid line represents variance of correlations and the dashed line represents FDR(c). For comparison, we transform $var(\rho_{ij})$ to $var(\rho_{ij})/10{+}0.1$ so that two quantities have same scale.
